# Translation and evaluation of psychometric properties of the Persian version of the Hypertension Self-Care Activity Level Effects (H-SCALE)

**DOI:** 10.1186/s12872-023-03460-z

**Published:** 2023-08-26

**Authors:** Mohammadamin Shabani, Zahra Taheri-Kharameh, Fatemeh Sheikholeslamikabiri, Maede Judy

**Affiliations:** 1 Students Research Committee, Qom University of Medical Sciences, Qom, Iran; 2 Spirituality Health Research Center, School of Health and Religion,, Qom University of Medical Sciences, Qom, Iran; 3 Department of Public Health, School of Health,, Qom University of Medical Sciences, Qom, Iran

**Keywords:** Psychometric, Hypertension, Self-care, Validity, Reliability

## Abstract

**Background & Objectives:**

Hypertension is a major cause of cardiovascular disease and premature death worldwide. Managing hypertension through self-care practices is considered one of the most effective strategies. However, to accurately assess the self-care status of individuals with hypertension, a valid and reliable tool is necessary. This study aimed to evaluate the psychometric properties of the Persian version of the Hypertension Self-Care Activity Level Effects Questionnaire.

**Methods:**

Present methodological study was conducted on 218 patients with hypertension visiting the Clinic of Qom educational and medical centers. Subjects were selected with convenience sampling. Data were collected using the H-SCALE, and a demographic questionnaire. The translation was done from the recommended backward-forward method according to the WHO protocol. After that, face and content validity were applied, along with construct validity involving a comparison of known groups and confirmatory factor analysis. To test reliability, Cronbach's alpha was used. Data analysis was performed by SPSS and smart-PLS software.

**Results:**

The factor loadings of the self-care behaviors questionnaire were significant in all constructs, and were higher than 0.5 except for three items. The known-groups method showed that the self-care score of patients with controlled blood pressure was significantly higher than those with uncontrolled blood pressure. The extent of average variance extracted (AVE) of the majority of the constructs in the questionnaire was greater than 0.5 and, the composite reliability was greater than the AVE, indicating a good convergent validity. The divergent validity of the questionnaire was confirmed using the Fornell-Larcker criterion and the heterotrait-monotrait ratio (HTMT). Cronbach's alpha coefficient, combined reliability, and communalities index were optimal.

**Conclusion:**

According to research findings, the Persian version of the questionnaire has good validity and reliability that can be used as a tool to measure the level of self-care of hypertension by health care providers.

## Introduction

The number of people with hypertension increased from 648,000,000 people in 1990 to 1278,000,000 in 2019, and the number of people with hypertension is projected to reach 1560,000,000 people 2025 [1]. A systematic review and meta-analysis conducted on the prevalence of hypertension in Iran from 2004 to 2018 showed a rate of 25% [2]. In a different study, the prevalence of stage 1 and stage 2 hypertension among Iranian adults was reported as 29.6% and 18.6%, respectively [3]. Hypertension is a major cause of cardiovascular disease and premature death worldwide [4]. High systolic blood pressure (SBP) was the main risk factor in the DALY (disability-adjusted life years) ranking, accounting for 10.4 million deaths and 218 million DALYs [5]. Risk factors for hypertension include high sodium intake, low potassium intake, obesity, alcohol consumption, physical inactivity, unhealthy diet, as well as genetics [4, 6]. Self-care is one of the most effective ways to deal with hypertension that helps people with hypertension to take more responsibility for their health [7]. Self-care includes the steps people take to lead a healthy lifestyle, take care of their chronic illness, and prevent further illness. In hypertension, the self-care behaviors recommended for optimal disease control include: (a) adherence to antihypertensive drugs, (b) following a healthy low-salt diet, (c) engaging in adequate physical activity, (d) quitting smoking and (e) moderating alcohol consumption [8, 9]. There is ample evidence that people with hypertension have poor adherence to their self-care behaviors [10]. For example, despite increasing public awareness and improved access to new drugs, only 30 to 40 percent of patients reported taking their medications regularly [11].

The evaluation of self-care practices in individuals with hypertension can provide valuable insights for healthcare professionals in managing this condition. By utilizing a standardized and efficient assessment of self-care practices, experts can offer guidance to encourage favorable behaviors and discourage risky ones, ultimately reducing the associated negative outcomes. However, the absence of reliable measurement tools for self-care can be a contributing factor to inadequate self-care among individuals with hypertension [12]. Morisky Medication Adherence Scale (MMAS-8) is one of the self-care scales in patients with hypertension [13], which is not comprehensive and only examines adherence to the medication regimen. The reliability and validity of this scale was reported as acceptable for patients with hypertension in the Iranian population [14]. Another scale is Hypertension self-care profile (HTN-SCP), the validity and reliability of which has been evaluated in Iran by Ghani Gheshlagh et al. with good validity and reliability, but this scale does not pay attention to the physical activity factor As an important factor in self-care [12].

The H-SCALE is a 31-item scale that developed by Warren-Findlow, and assesses all aspects of self-care in patients with hypertension [15]. Some studies have confirmed that the H-SCALE is a valid and reliable scale for the measurement of self-care in patients with hypertension living in different cultures and contexts [16, 17]. Although the English and Spanish versions of the H-SCALE have been psychometrically evaluated, there is a need for localized and comprehensive scales to assess self-care in patients with hypertension. Therefore, this study aimed to translate the H-SCALE into Persian and evaluate its psychometric properties in hypertensive patients in Iran, recognizing the importance of self-care in managing hypertension.

## Material and methods

### Participants and setting

This methodological study aimed to translate and evaluate the psychometric properties of the H-SCALE questionnaire. The study participants were patients with hypertension who were referred to the Qom educational and medical center clinic in Iran between September 2021 and April 2022. The convenience sampling method was used to select participants for the study. Inclusion criteria were age equal to and over 18 years, diagnosis of hypertension by a cardiologist, no mental or cognitive impairment, at least 6 months after diagnosis of disease, ability to communicate and respond, and consent to participate in the study. The minimum required sample size for factor analysis depends on several factors, such as the complexity of the data and the number of variables being analyzed. However, a widely accepted rule of thumb is to have at least five participants per variable being analyzed [18]. Two hundred and eighteen patients were included in the study according to the criteria after obtaining permission from the university Vice Chancellor for Research and coordination with educational and medical centers. Then, the purpose of the project and how to do it was explained to the participants. The questionnaires were completed within 6 months after ensuring the confidentiality of the information with the researcher and obtaining their consent.

### Measures

To collect data, H-SCALE, and demographic and medical information were used:

The H-SCALE questionnaire comprises six subscales, which are medication adherence, weight management, physical exercises, smoking exposure, alcohol intake, and healthy eating plan. The medication subscale includes three questions that assess drug adherence in the past 7 days, with each question scored from 0 to 7 and a total score range of 0 to 21 points. A score of 21 indicates a positive attitude towards medication adherence. The healthy eating plan subscale consists of 10 questions that assess healthy eating habits, with seven of these questions reverse-scored. Each question is scored from 0 to 7, resulting in an overall score range of 0 to 70. A score of 6 out of 7 is considered a positive score for each question, and a high score of 60 indicates a positive score for the subscale. The physical activity subscale includes two questions that assess physical activity levels, with each question scored from 0 to 7 and a total score range of 0 to 14. A score higher than 8 indicates positive behavior towards physical activity. The smoking exposure subscale includes two questions that assess smoking exposure, with each question scored from 0 to 7 and a total score range of 0 to 14. A score of zero on both questions indicates a positive attitude towards smoking exposure. The weight Management subscale comprises nine questions that measure weight control behaviors in the past month. Each question is scored on a 0 to 5 Likert scale, with a total score range of 9 to 45. A score above 35 is considered positive in weight management. Alcohol intake was assessed using three items, with patients who did not consume any alcohol considered abstainers [15].

The sociodemographic form included the following variables: age, gender, residence status, marital status, employment status, level of education, level of income, duration of illness, comorbidiy, history of smoking, and body mass index (BMI). Additionally, participants were asked to self-report whether their blood pressure was under control or not.

### Translation and validation of Persian version

To perform the translation process, the recommended backward-forward method based on the protocol of the International Quality of Life Assessment Project (IQoLAP) was used [19]. For this purpose, first, 2 fluent English translators performed 2 separate translations of the English version of the questionnaire into Persian. The original Persian version of the above two translations was obtained by considering the better translation. Next, two English language experts re-translated the final version into English. After this stage, the original English version was compared with the English version obtained by the translation of language experts by the research team, and finally, with the necessary corrections and editing, the final Persian version was approved. Any discrepancies were discussed and resolved through consensus, taking into account the cultural and linguistic nuances of the Persian language.

To evaluate the face validity of the scale, the writing style, wording, and overall appearance were assessed for their logical and engaging presentation. To accomplish this, the questionnaire was administered to 10 patients who met the inclusion criteria. They were asked to provide feedback on the content, clarity, eligibility, simplicity, and ease of understanding of the instrument terms, as well as the ease of completing the questionnaire.

During this evaluation, the level of difficulty (difficulty in understanding phrases and words), appropriateness (relationship of phrases with the dimensions of the questionnaire), and ambiguity (potential misunderstandings of phrases or deficiencies in the meanings of words) were examined. In addition, feedback from content experts was used to enhance the face validity of the scale.

For this purpose, five experts in the relevant field were asked to provide feedback after conducting a qualitative review of the questionnaire based on criteria such as grammar, use of appropriate words, necessity, importance, placement of phrases in their proper place, and proper scoring. The opinions of the older participants and the content experts were used to refine the scale, ensuring that it was both valid and appropriate for use in the target population.

To assess the construct validity of the questionnaire in this study, the Known Group Comparison technique was utilized. This technique is used to determine the extent to which the questionnaire can differentiate between different subgroups. Specifically, this type of validity determines the ability of a scale to differentiate respondents based on predetermined criteria and assumptions.

In this study, disease control was used as the parameter. The scale score was compared between two groups of patients, those with and without blood pressure control, using an independent t-test. We hypothesized that individuals with controlled disease would score higher on the questionnaire than patients without controlled blood pressure.

The validity of the questionnaire was assessed using Confirmatory Factor Analysis (CFA), which examined both convergent validity and divergent validity. Convergent validity was measured using factor loads, Average Variance Extracted (AVE), and composite reliability. A factor load of 0.6 or greater was required to establish convergent validity, along with an AVE greater than 0.5 and composite reliability greater than the AVE. Divergent validity was assessed using cross loading, the Fornell-Larcker test, and the Heterotrait-Monotrait Ratio (HTMT). Cross loading required each question to have a factor load on its own construct greater than the factor load on other constructs, with a difference of at least 0.1. The Fornell-Larcker test required the root mean square of the extracted variance of each variable to be greater than the maximum correlation of that variable with other variables. The HTMT index was also used to assess divergent validity, with values below 0.9 indicating construct validity of the questionnaire. These methods provided a comprehensive assessment of the validity of the questionnaire [20].

Internal consistency, composite reliability, and communalities are the main criteria used to assess the reliability of the questionnaire. Cronbach's alpha above 0.6, the composite reliability above 0.7 and the communalities index above 0.5 are confirmed [21]. The Cronbach's alpha coefficients were used to assess internal consistency reliability, which indicates the extent to which individual items in a construct measure the same underlying concept. The composite reliability was used to assess the reliability of the constructs as a whole, which takes into account both the internal consistency of the items and the amount of variance in the construct that is accounted for by the measurement.

## Results

The mean age of participants was 58.34(SD = 10.3) years. The majority of participants were females (68%) and their education level 40% were illiterate or elementary. Also, 84% of the participants were married and 75.5% of them owned housing. The average duration of the disease was 8.85 years (SD = 6.42), with a range of 1 to 30 years.

In order to evaluate the known group comparison by the Persian version of the questionnaire based on disease control, independent t-test was used (Table 1).

**Table 1:** Known Groups Comparison: mean score of self-care behaviors in patients with and without controlled hypertension

Variable or H-SCALE subscale	Controlled hypertension (Standard deviation) average *n* = 157	Uncontrolled hypertension (Standard deviation) average *n* = 37	*P*-value
Medication adherence	20.16 (2.51)	16.16 (5.30)	0.003
Healthy eating plan or Low-salt Diet	33.32 (11.22)	31.81 (9.53)	0.041
Physical Activity	3.96 (4.45)	3.86 (3.81)	0.431
Smoking exposure	1.15 (1.92)	2.08 (2.36)	0.152
Weight management	23.65 (8.92)	22.18 (9.66)	0.501

The results of the Known Groups Comparison analysis indicate that there was a significant difference in the mean scores of the questionnaire between patients who had controlled blood pressure and those who did not. Specifically, patients who had controlled blood pressure had a significantly higher mean score on the questionnaire for the medication use construct (*P* = 0.003) and the healthy eating plan construct (*P* = 0.041) compared to patients who did not have controlled blood pressure.

**Fig. 1:**
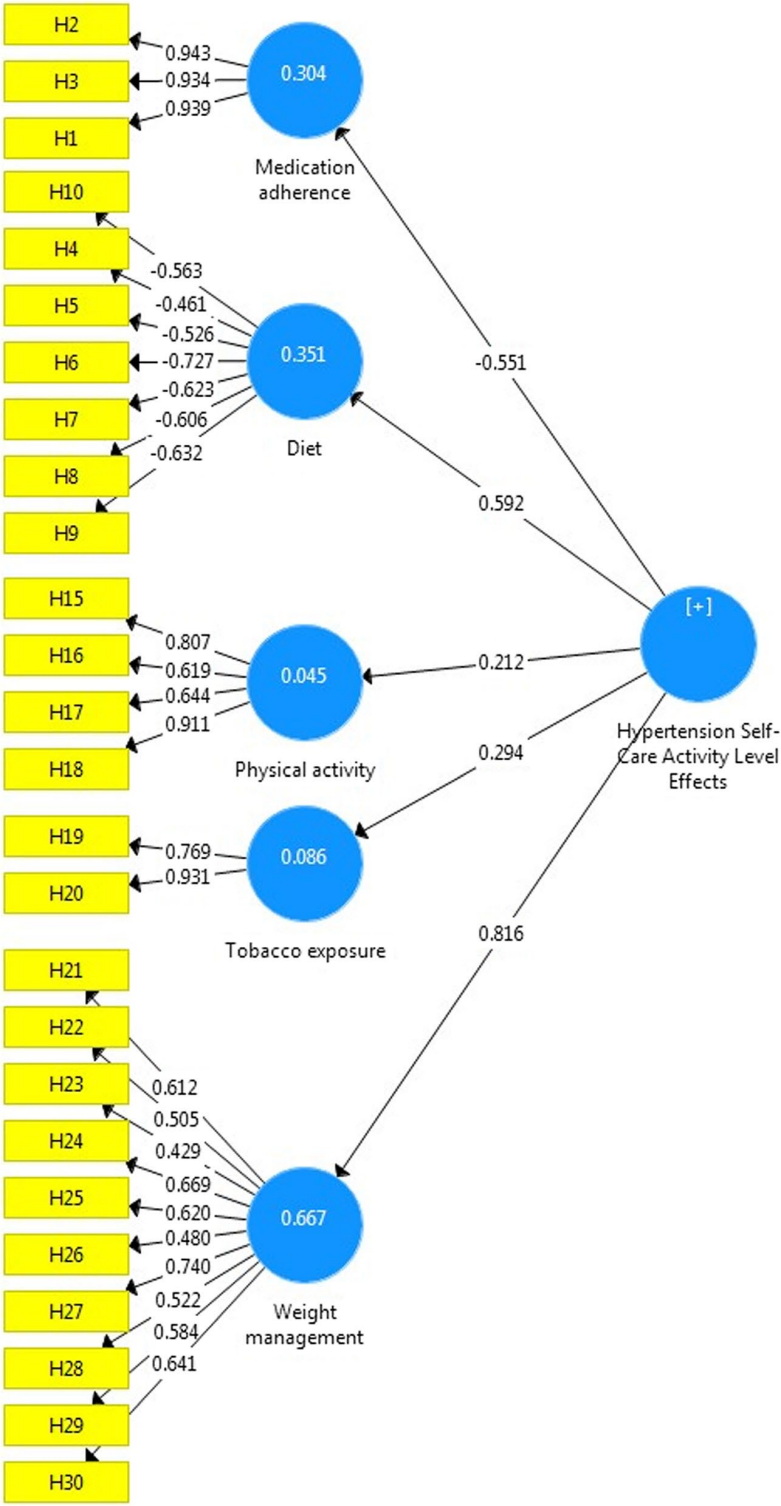
Confirmatory Factor Analysis of the Persian version of the Hypertension Self-Care Activity Level Effects (H-SCALE)

The factor loadings of the questionnaire were the effects of the level of self-care activity in all constructs and were higher than 0.5 except for three items (Fig. 1).

Based on the results presented in Table 2, the mean extracted variance of the constructs, excluding the healthy eating plan and weight management constructs, was greater than 0.5. Additionally, the composite reliability of all variables was greater than the mean extracted variance, indicating that most of the questionnaire constructs had convergent validity. In addition, Table 2 showed the Cronbach's alpha coefficients, composite reliability and communalities index of all constructs. The Cronbach's alpha coefficients ranged from 0.65 to 0.93, which indicates good internal consistency for most of the constructs. The composite reliability values ranged from 0.79 to 0.97, which indicates good overall reliability for all constructs.

**Table 2:** Mean values of extracted variance and reliability indices of hypertension self-care behavior questionnaire

Variable	Average Variance Extracted (AVE)	Composite reliability	Cronbach's alpha	Communalities
Medication adherence	0.888	0.975	0.933	0.956
Healthy eating plan	0.356	0.792	0.695	0.688
Physical activity	0.570	0.837	0.846	0.291
Smoking exposure	0.729	0.842	0.652	0.824
Weight management	0.445	0.837	0.748	0.796

The results of Table 3 showed that the mean of extracted variance of all variables was obtained from the correlation of that variable with other larger variables. Therefore, divergence validity at the level of study construct was ensured.

**Table 3:** Divergent validity: Fornell-Larker index of hypertension self-care behavior questionnaire constructs

Variable	1	2	3	4	5
1. Medication adherence	0.596				
2. Healthy eating plan	-0.101	0.938			
3.Physical activity	0.060	-0.159	0.755		
4. Smoking exposure	0.024	-0.100	0.132	0.854	
5.Weight management	0.266	-0.205	0.043	0.197	0.587

Also, the results of Table 4 showed that the HTMT index in all constructs is less than 0.9, so the divergent validity of the questionnaire was confirmed.

**Table 4:** Divergent validity—HTMT index of hypertension self-care behavior questionnaire constructs

Variable	1	2	3	4
1. Healthy eating plan				
2. Medication adherence	0.175			
3.Physical activity	0.140	0.117		
4. Smoking exposure	0.182	0.117	0.125	
5.Weight management	0.383	0.240	0.200	0.256

## Discussion

The results of the study indicate that the Persian version of the H-SCALE is a reliable and valid tool for measuring self-care behaviors related to hypertension in Persian-speaking populations.

In this study, the translation of the questionnaire was conducted with great care by individuals who were fluent in both the source and target languages, and knowledgeable about the subject matter. The translation process followed established principles and guidelines, and particular attention was paid to ensuring accuracy and cultural adaptation of the questionnaire. As a result of cultural and religious norms prevalent in Iran, and the absence of any reported alcohol use among the participants, the item concerning alcohol consumption was eliminated from the questionnaire. This was done in order to ensure that the questionnaire was culturally appropriate and relevant to the study population. The elimination of this item did not compromise the validity or reliability of the questionnaire for the purposes of the study.

The study reported good convergent validity, as the AVE of the constructs, excluding the healthy eating plan and weight management constructs, was greater than 0.5. Additionally, the composite reliability was greater than the AVE, indicating that most of the questionnaire constructs had convergent validity. The study also confirmed the divergent validity of the questionnaire using both the Fornell-Larker index and the HTMT index. The Fornell-Larker index showed that the mean of extracted variance of all variables was obtained from the correlation of that variable with other larger variables, indicating good divergent validity. The HTMT index also confirmed the divergent validity of the questionnaire, as all constructs had an index value less than 0.9. Likewise, in a study conducted by Warren-Findlow et al. (2020), the questionnaire was found to have good construct validity [22].

Furthermore, the study used the known-groups method to evaluate construct validity, based on blood pressure control. The analysis showed that the scale score of patients with controlled blood pressure was significantly higher than those with uncontrolled blood pressure. This indicates that the H-SCALE is able to distinguish between patients with controlled and uncontrolled hypertension, and can be used to identify areas where patients need to improve their self-care behaviors. These findings are consistent with the original study by Warren-Findlow et al., which also showed a significant relationship between blood pressure control and H-SCALE scores [15]. Similarly, a study by Gusty et al. (2017) found that there was a significant correlation between the H-SCALE and blood pressure monitoring [23].

The internal consistency of the Persian version of the H-SCALE was found to be good for most constructs, with Cronbach's alpha coefficients ranging from 0.65 to 0.93. The composite reliability values also indicated good overall reliability for all constructs, ranging from 0.79 to 0.97. These findings are consistent with the main study and suggest that the Persian version of the H-SCALE is a reliable tool for measuring self-care behaviors related to hypertension in Persian-speaking populations.

Similar results were reported in a study by Warren-Findlow et al. (2013) that evaluated the psychometric properties of the H-SCALE in an English-speaking population. In that study, the Cronbach's alpha coefficients for the subscales were medication: 0.77, DASH diet: 0.67, physical activity: 0.77, smoking exposure: 0.78, weight management: 0.86, and Alcohol intake: 0.88. The internal consistency of all subscales, except for the DASH diet subscale, was found to be desirable [16]. In a subsequent study by Warren-Findlow et al. (2018), which also evaluated the psychometric properties of the H-SCALE among Hispanics, the Cronbach's alpha coefficients for the subscales ranged from 0.75 to 0.91. These values indicate acceptable to good internal consistency, consistent with the findings of the current study on the Persian version of the questionnaire [17].

One limitation of the study is the non-random sampling method used, which may restrict the generalizability of the study findings beyond the study population. Further studies with larger sample sizes and multi-state studies are recommended to improve the generalizability of the findings. Another limitation is that the study only evaluated the validity and reliability of the H-SCALE, without assessing its responsiveness. More studies are needed to assess the responsiveness of the H-SCALE to changes in self-care behaviors related to hypertension over time. Also, in future studies, it is recommended to examine criterion validity using standard self-care tools.

Despite these limitations, a strength of the study is that it provides evidence for the validity and reliability of the Persian version of the H-SCALE, which can be used to measure self-care behaviors related to hypertension in Persian-speaking populations.

## Conclusion

The study demonstrated that the H-SCALE questionnaire has good psychometric properties for measuring blood pressure self-care level and healthcare outcomes in various clinical and research settings. These findings have important implications for clinical practice, as the H-SCALE can be used to identify areas where patients need to improve their self-care behaviors and develop tailored interventions to improve hypertension management.

## Data Availability

The datasets used and/or analysed during the current study available from the corresponding author on reasonable request.

## Abbreviations

H-SCALE: Hypertension Self-Care Activity Level Effects
SBP: Systolic blood pressure
DALY: Disability-adjusted life years
HTN-SCP: Hypertension self-care profile
IQoLAP: International Quality of Life Assessment Project
HTMT: Heterotrait-Monotrait
